# Supplementary material for: Synergy of Small Antiviral Molecules on a Black-Phosphorus Nanocarrier: Machine Learning and Quantum Chemical Simulation Insights
Source: Molecules. 2023 Apr 17;28(8):3521. doi: 10.3390/molecules28083521 (PMC10142408; doi:10.3390/molecules28083521)
Supplement: Supplementary file 1 [file molecules-28-03521-s001.zip › molecules-2309552-supplementary.pdf]

Supplementary Materials

Synergy of Small Antiviral Molecules on a Black-Phosphorus Nanocarrier: Machine Learning and Quantum Chemical Simulation Insights

Slimane LAREF[*,1], Fouzi Harrou[*,2], Bin WANG[3], Ying Sun[2], Amel Laref[4], Meriem Laleg–Kirati[2], Takashi GOJOBORI[1], and Xin GAO[1]

[1]Computational Bioscience Research Center (CBRC), King Abdullah University of Science & Technology (KAUST), Thuwal 23955-6900, Kingdom of Saudi Arabia.

[2]Computer, Electrical and Mathematical Sciences and Engineering (CEMSE) Division, King Abdullah University of Science & Technology (KAUST), Thuwal 23955-6900, Kingdom of Saudi Arabia.

[3]School of Chemical, Biological and Materials Engineering, Center for Interfacial Reaction Engineering (CIRE), University of Oklahoma, Norman, Oklahoma 73019, United States.

[4]Department of Physics and Astronomy, College of Science, King Saud University, Riyadh 11451, Saudi Arabia.

Fouzi HARROU, Bin WANG, Ying SUN, Amel Laref, Meriem LALEG-KIRATI, Takashi GOJOBORI, and Xi GAO contributed equally to this work.

*Corresponding authors. Emails: slimane.laref@kaust.edu.sa and Emails: fouzi.harrou@kaust.edu.sa

The interaction of favipiravir ($C_5H_4FN_3O_2$) and ebselen ($C_{13}H_9NOSe$) molecules on black–phosphorus (BP) is studied on the most energetically stable configurations. To locate the most stable interaction energy or non-bond energy, the surface into a grid of (1×1) 0.25 Å is considered. The interaction of the molecules at each grid–site is examined throughout structural relaxation at T = 0 K, and the configuration corresponding to the lowest interaction energy geometry is selected. Frequently, there are many favorable sites that are available for adsorption of drugs onto the two–dimension (2D) surfaces; in this study, we retained only the geometry of the lowest energy levels.

The non–bond energy, $\Delta E_{Non-Bond}$ of drugs on the surface is calculated as follows:

$$\Delta E_{Non-Bond} = E_{Tot} - (E_{BP} + E_{Mol})$$

E_{BP} is the unrelaxed energy of the surface, E_{Mol} is the unrelaxed isolated energy of the molecule, and E_{Tot} is the relaxed energy of the surface and the adsorbed molecule. A negative $E_{Non-Bond}$ signifies stable adsorption. For simplicity, we refer only to the numerical values of non–bond energy in the entire manuscript. It is noted that we restrict the calculations to characterizing

the interaction of the molecules on the respective surface. Consequently, the reported non-bond energies are in units of eV per molecule (or kcal/mol). The comprehensive interaction details of these drugs with black–phosphorus are presented in the main paper.

Table S1: ML, and PBE–D3(BJ) non–bond energy prediction of MLFF–EB, MLFF–FP, and MLFF–FP_EB adsorption states in (eV) and (kcal/mol) at T = 0 K.

	MLFF–FP_EB	MLFF–FP	MLFF–EB
ML(eV)	-2.00	-0.68	-1.26
PBE–D3(eV)	-2.15	-0.82	-1.31
ML(kcal/mol)	-46.03	-16.00	-29.00
PBE–D3(kcal/mol)	-49.60	-18.45	-30.12

Table S2: ML, and PBE–D3(BJ) van der Waals energy prediction of MLFF–EB, MLFF–FP, and MLFF–FP_EB adsorption states in (eV) and (kcal/mol) at T = 0 K.

	MLFF–FP_EB	MLFF–FP	MLFF–EB
ML(eV)	-2.25	-0.75	-1.33
PBE–D3(eV)	-2.36	-0.79	-1.43
ML(kcal/mol)	-51.89	-17.30	-30.67
PBE–D3(kcal/mol)	-54.42	-18.22	-32.98

Ideally, the aromatic molecules are released from the surface at the external stimulus (by adding charge) or such as rising temperatures; this process is simulated by calculating the Gibbs free energy of the complex with and without the drug. The Gibbs free energy changes for all intermediates configuration during the loading and releasing process are calculated as follow:

$$\Delta G = \Delta E_{DFT} + \Delta E_{ZPE} + \text{T } \Delta S$$

where ΔE_{DFT}, ΔE_{ZPE}, and T ΔS represent the difference of DFT–calculated binding energy, zero–point energy (ZPE), and entropy contribution (S), respectively.

*The data and results associated with this article are available at https://github.com/SLIM23-CBRC/MD-ML-Hamiltonian.

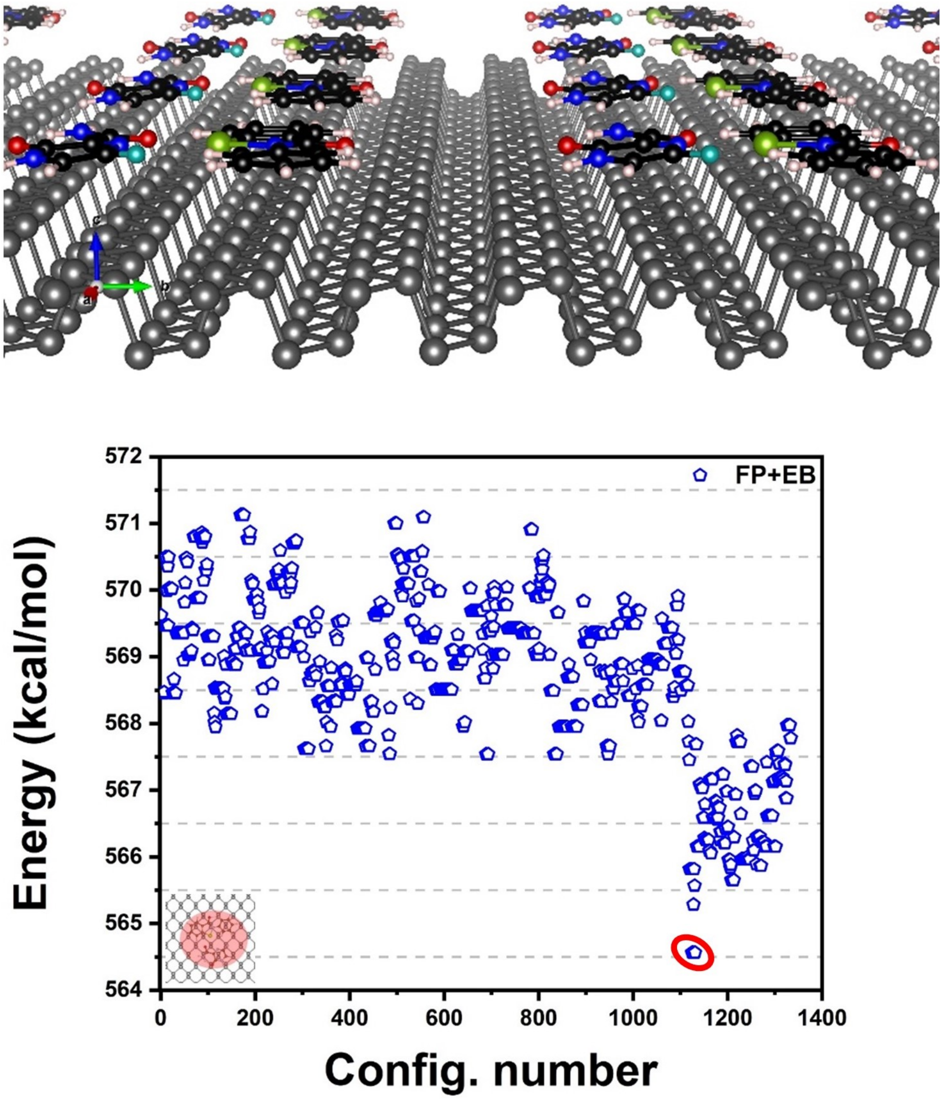

Figure S1: Molecular dynamics simulations potential energy prediction: (**a**) snapshot of FP-EB adsorption molecule on Black-Phosphorus, and (**b**) potential energy change of FP-EB on BP surface.
